# Reduction of Baltic Sea Nutrient Inputs and Allocation of Abatement Costs Within the Baltic Sea Catchment

**DOI:** 10.1007/s13280-013-0484-5

**Published:** 2014-01-12

**Authors:** Fredrik Wulff, Christoph Humborg, Hans Estrup Andersen, Gitte Blicher-Mathiesen, Mikołaj Czajkowski, Katarina Elofsson, Anders Fonnesbech-Wulff, Berit Hasler, Bongghi Hong, Viesturs Jansons, Carl-Magnus Mörth, James C. R. Smart, Erik Smedberg, Per Stålnacke, Dennis P. Swaney, Hans Thodsen, Adam Was, Tomasz Żylicz

**Affiliations:** 1 Department of Ecology, Environment and Plant Sciences, Baltic Nest Institute (BNI), Stockholm University, 106 91 Stockholm, Sweden; 2 Department of Bioscience, Aarhus University, 8600 Silkeborg, Denmark; 3 Warsaw Ecological Economics Center, University of Warsaw, Dluga 44/50, 00-241 Warsaw, Poland; 4 Department of Economics, Swedish University of Agricultural Sciences, 750 07 Uppsala, Sweden; 5 Department of Environmental Science, Aarhus University, 4000 Roskilde, Denmark; 6 Department of Ecology and Evolutionary Biology, Cornell University, Ithaca, NY 14853 USA; 7 Department of Applied Environmental Science and Baltic Nest Institute, Stockholm University, 106 91 Stockholm, Sweden; 8 Department of Environmental Engineering and Water Management, Latvia University of Agriculture, 19 Akademijas Str., Jelgava, 3001 Latvia; 9 Department of Geological Sciences, Stockholm University, 106 91 Stockholm, Sweden; 10 Griffith School of Environment, Griffith University, South Brisbane, QLD 4111 Australia; 11 Baltic Nest Institute, Stockholm University, 106 91 Stockholm, Sweden; 12 Department of Water Quality and Hydrology, Norwegian Institute for Agricultural and Environmental Research (Bioforsk), 1432 Ås, Norway; 13 Institute of Bioscience, Aarhus University, 8600 Silkeborg, Denmark; 14 Faculty of Economic Sciences, Warsaw University of Life Sciences, Dluga 44/50, 00-241 Warsaw, Poland; 15 Faculty of Economic Sciences, University of Warsaw, Dluga 44/50, 00-241 Warsaw, Poland

**Keywords:** Nutrient reduction, Nitrogen, Phosphorus, Retention, Management, Cost minimization

## Abstract

The Baltic Sea Action Plan (BSAP) requires tools to simulate effects and costs of various nutrient abatement strategies. Hierarchically connected databases and models of the entire catchment have been created to allow decision makers to view scenarios via the decision support system NEST. Increased intensity in agriculture in transient countries would result in increased nutrient loads to the Baltic Sea, particularly from Poland, the Baltic States, and Russia. Nutrient retentions are high, which means that the nutrient reduction goals of 135 000 tons N and 15 000 tons P, as formulated in the BSAP from 2007, correspond to a reduction in nutrient loadings to watersheds by 675 000 tons N and 158 000 tons P. A cost-minimization model was used to allocate nutrient reductions to measures and countries where the costs for reducing loads are low. The minimum annual cost to meet BSAP basin targets is estimated to 4.7 billion €.

**Electronic supplementary material:**

The online version of this article (doi:10.1007/s13280-013-0484-5) contains supplementary material, which is available to authorized users.

## Introduction

The Baltic Sea has suffered from severe effects of eutrophication for many decades. The Baltic Sea Action Plan (BSAP) of the Helsinki Commission (HELCOM) was adopted by all the coastal countries of the Baltic Sea and by the European Community in November 2007 (HELCOM 2007). The eutrophication section of the BSAP is commonly considered as its most important component, since it presents very specific goals in terms of nutrient reductions (in tons of nitrogen and phosphorus) for the various sub-basins in order to achieve a “healthy” Baltic by 2021 (Backer et al. 2010). Moreover, these nutrient reduction goals are allocated to the countries around the sea. Models and datasets covering the entire sea and catchment were used in these calculations (Wulff et al. 2007).

The novelty of the approach used in the HELCOM action plan (BSAP) is that it puts the ecosystem at the center, defining the status of the sea as we want it to be in the future, and focusing management decisions on this goal instead of taking the traditional approach of addressing pollution sources on a sector-by-sector basis, without directly linking abatement measures to the status of the Baltic Sea (Pyhälä 2012).

When the BSAP was adopted, it was recognized that the calculated maximum allowable nutrient loads and the country-wise allocations of nutrient reductions were based on the best knowledge available, but that revised estimates would be necessary as soon as updated data and more advanced models became available. These revisions have now been made (late fall 2013), but have not yet been approved by all HELCOM member countries.

The economic cost of implementing nutrient reductions is not addressed in the BSAP, but is estimated to be high (Elofsson 2010a). Policymakers are likely to be concerned with the costs incurred within their respective countries, and well-founded estimates of nutrient reduction costs and their distribution could serve as a basis for negotiations among countries as well as for the selection and design of economic incentives.

The BONUS research project RECOCA (*Re*duction of Baltic Sea Nutrient Inputs and *Co*st Allocation in the Baltic Sea *Ca*tchment) was specifically designed to improve our understanding of processes in the catchment, compared to those used in the original BSAP, by using improved models and datasets. The key objectives of RECOCA were to (1) simulate possible future riverine nutrient loads to the Baltic Sea, (2) estimate cost-effective reductions of those loads, and (3) suggest cost allocation schemes for the countries in the drainage basin. In this paper, we describe a multi-model approach to characterize the nutrient loads, the retentions that occur between these sources and the Sea, and the effects of various management strategies to reduce loads. An advantage of the approach, in which models of different levels of complexity and spatial resolution are applied to the basin (see Electronic Supplementary Material, Fig. S1), is that it provides more robust insights into patterns of loading and response when the models yield similar results and provides insight into priorities for additional research when they disagree.

## Key Research and Results

### New Catchment Database

**Fig. 1:**
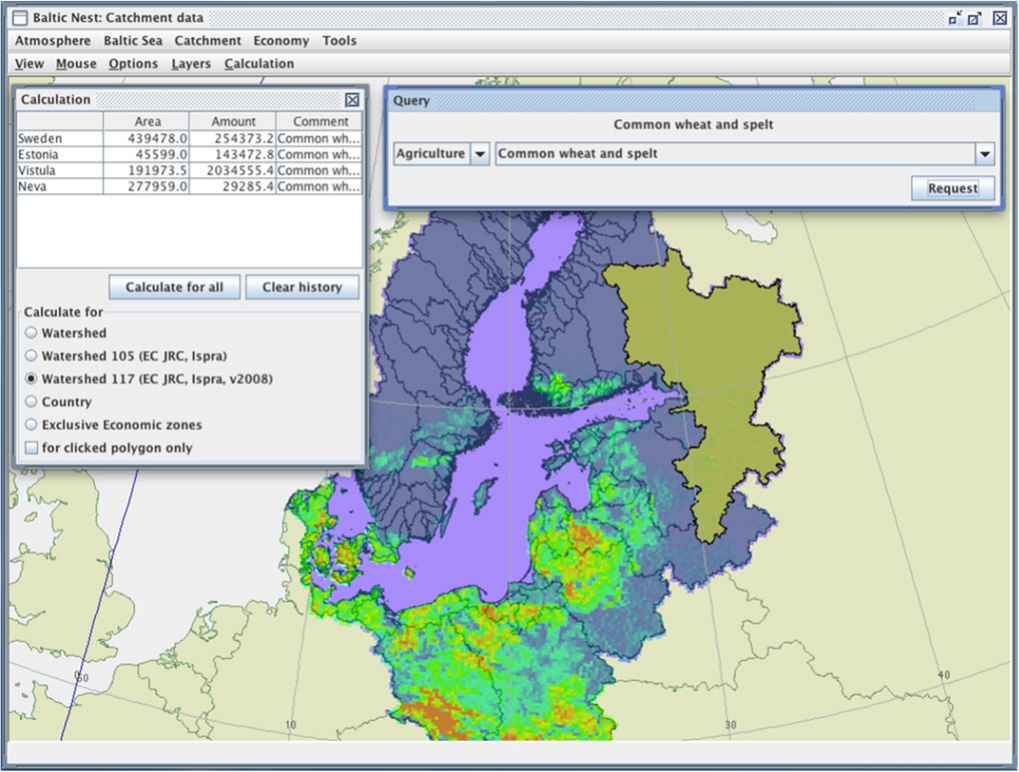
The new catchment database accessible via the decision support system Nest (www.balticnest.org). This example shows agricultural data, specifically the distribution of cultivation of common wheat and spelt. The Nest interface allows the user to make various calculations, in this case aggregate data for countries or sub-catchments

We have assembled datasets from many sources and then compiled gridded data with high spatial resolution over the entire Baltic watershed. These can then be used in watershed-scale nutrient accounting tools and models. These gridded data are now available via the Nest decision support system (Fig. 1). Data sources include the EU Joint Research Centre (fertilizer use, crop types), EUROSTAT (livestock data), HYDE database (population), CORINE (land cover), and SMHI (hydrological and climate forcing). For further details, see Hong et al. (2012). These data have been compiled for all the 117 watersheds that comprise the Baltic Sea drainage area (82 major watersheds and 35 coastal areas) as well as for 8 “type watersheds,” and are organized into fertilizer use, atmospheric deposition, biological N-fixation, crops, livestock, and human population distributions.

Furthermore, data from EUROSTAT and the EU Farm Accounting Data Network were used to estimate costs of reducing livestock production, fertilizer inputs, and changing land use. A detailed description of the distributions of point sources of nutrient pollution, i.e., municipal wastewater treatment (WWT) systems, including estimates of the populations connected and not connected to sewage systems, was also created (Table 1).

**Table 1:** An example of datasets compiled for the Baltic Sea catchment (Hong et al. 2012)

Item	Bothnian Bay	Bothnian Sea	Gulf of Finland	Gulf of Riga	Kattegat	Baltic Proper	Danish Straits
Area (km^2^)	269 576	230 953	418 980	136 179	90 081	573 368	27 357
Population density (persons km^−2^)							
Total	4.9	11.5	26.7	27.7	36.2	94.5	172.7
Urban	3.2	8.1	18.9	18.5	29.4	61.5	148.2
Rural	1.7	3.4	7.8	9.2	6.8	33.0	24.6
Livestock density (animals km^−2^)							
Cattle	1.1	1.5	2.6	7.4	12.2	13.1	32.1
Pigs	1.0	2.7	3.3	8.3	62.5	37.5	150.9
Poultry	30.0	28.3	130.4	73.5	94.1	357.2	341.1
Sheep	0.1	0.4	0.2	0.5	2.0	1.3	6.9
Crop production (kg km^−2^ year^−1^)							
Barley	2047	4245	2248	5093	19 035	10 988	58 853
Wheat	63	1796	1204	6674	25 990	23 140	120 255
Maize (green)	0	0	2902	1883	8856	25 061	126 952
Oats	1120	2386	1202	1331	5578	3736	3759
Rye	20	140	177	1937	1683	9048	9237
Other cereal	32	238	68	1287	1909	13 573	4662
Potatoes	1344	1468	2908	12 709	8671	36 478	21 013
Rape and turnip	88	222	172	985	2146	3915	18 184
Sugar beet	103	3193	410	3831	6582	27 811	150 296
Fodder roots	0	0	21	1930	4071	6932	5966
Pulses	3	13	8	116	79	309	203
Leguminous plants	339	88	960	4653	360	6620	2577
Fruits and berries	17	24	67	469	223	5996	2499

### Nutrient Accounting Tools and Nutrient Retention in Catchments

Net Anthropogenic Nitrogen Inputs (NANI), first introduced by Howarth et al. (1996) for North Atlantic watersheds, represent human-induced nitrogen inputs to a watershed and have been shown to be a good predictor of riverine nitrogen export on a large-scale, multi-year average basis (Howarth et al. 2012). A corresponding approach for phosphorus (NAPI) accounts for major P inputs in a similar manner as with N, excluding terms for crop fixation, which do not exist for P, and atmospheric deposition, which is generally negligible (see Electronic Supplementary Material).

**Fig. 2:**
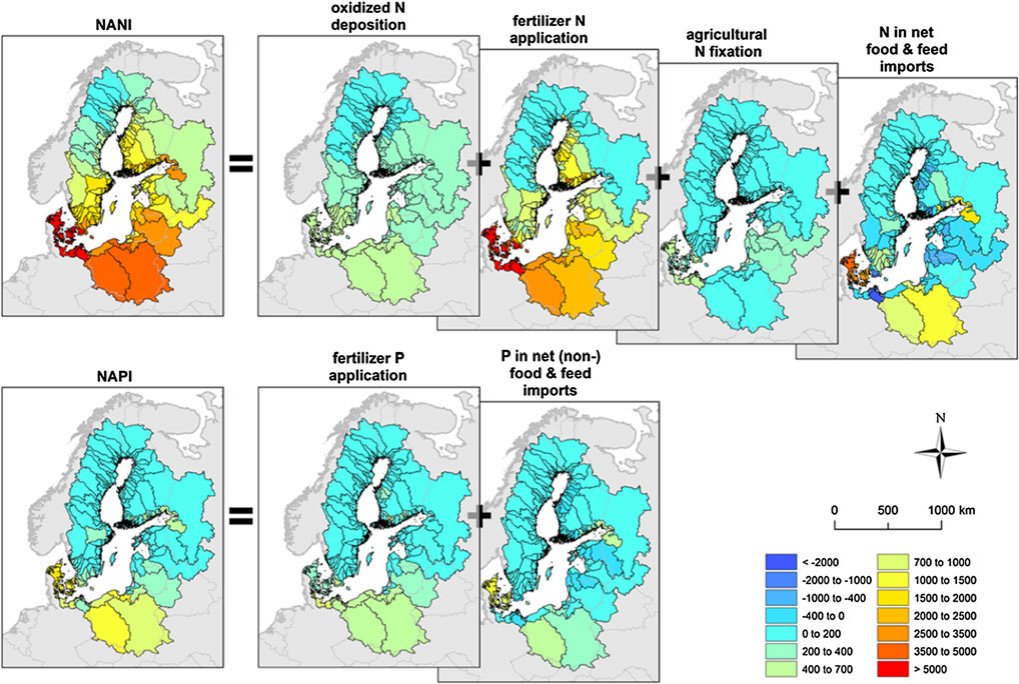
NANI (kg-N km^−2^ year^−1^) and NAPI (kg-P km^−2^ year^−1^), and their components in the Baltic Sea catchments (redrawn from Hong et al. 2012). The “P in net (non-)food & feed imports” includes human P consumption for both food and non-food use (e.g., detergents). *Positive numbers* mean net addition of nutrients to the catchments (e.g., import of food and feed), whereas *negative numbers* mean net removal of nutrients from the catchments (e.g., export of food and feed)

NANI, NAPI, and their components exhibited substantial variations among the Baltic Sea catchments (Fig. 2). Agricultural N-fixation in Baltic Sea catchments was estimated to be much lower than that in the US (Hong et al. 2011), reflecting relatively small areas of N-fixing crops in this region, unlike the US where soybean is one of the major crops. Nitrogen fluxes in net food and feed imports were often negative (i.e., positive net export of N as food or feed), although the magnitude of the negative values was again much smaller than in the US, for example, compared to the areas of the Corn Belt (Hong et al. 2011). Phosphorus fluxes generally showed a similar spatial pattern to nitrogen fluxes, although N fluxes were much higher in magnitude than the P fluxes.

Nutrient retention is the permanent removal or storage of nutrients and other biogenic elements within a system, i.e., the difference between nutrient inputs to a watershed and its riverine exports over the timescale considered (von Schiller et al. 2008). Conceptually, total retention within a catchment can be sub-divided into retention in soils, groundwater, and surface waters. Catchment processes contributing to retention are of vital importance for the economic evaluation performed in RECOCA (see below); retention is critical to finding a cost-optimal solution when the aim is to model cost-effective nutrient load reductions to the sea, as the effectiveness of abatement measures differs between source locations and target waters. Depending on the hydrological pathways, catchment retention processes may significantly alter elemental concentrations before they reach the sea (Stålnacke et al. 2003). For instance, wastewater treatment plants (WWTPs) discharge nutrients directly into surface waters, whereas agricultural nitrate losses normally leach from the root zone and are transported by groundwater to streams.

The differences between net anthropogenic inputs and observed riverine nutrient exports to the sea from catchments are an expression of catchment-scale retention. Nutrient loads and fluxes of water (average values for the period 1994–2006) were here taken from the ongoing HELCOM pollution load compilation PLC-5 (http://www.helcom.fi). The dataset covers almost 400 rivers and coastal regions, aggregated into 117 watersheds in which 78 major monitored rivers were identified, draining approximately 1 487 700 km^2^ (86 % of the total catchment area), as well as 29 coastal areas, draining approximately 227 800 km^2^ (13 %).

**Fig. 3:**
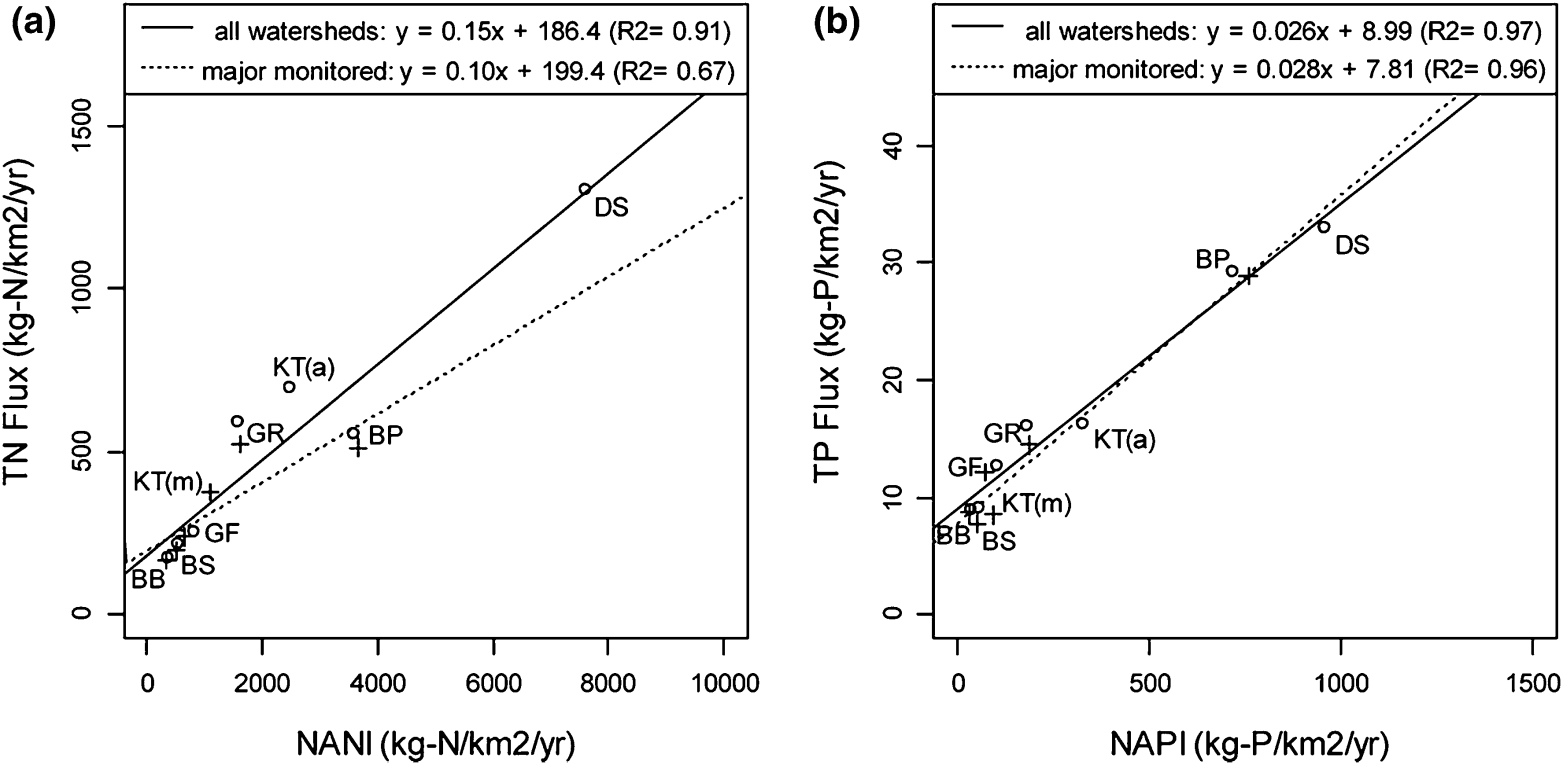
Relationships between NANI and riverine TN fluxes (**a**) and between NAPI and riverine TP fluxes (**b**) in seven regions of Baltic Sea catchments. NANI and NAPI are calculated with spatially uniform parameters. *Open circles* represent regional averages calculated from all watersheds with estimates of riverine TN and TP fluxes (107 watersheds); *plus symbols* from monitored watersheds only (78 watersheds). *BB* Bothnian Bay, *BS* Bothnian Sea, *GF* Gulf of Finland, *GR* Gulf of Riga, *KT* Kattegat, *BP* Baltic Proper, and *DS* Danish Straits. No monitored data were available in the DS region. Only the KT region showed a substantial difference between all watersheds and monitored watersheds only, and is thus separately labeled as “KT(a)” and “KT(m),” respectively

Riverine exports of N and P in the watersheds of Baltic Sea catchments correlate well to the NANI/NAPI loadings, with *R* ^2^ values between 0.66 and 0.97 (Fig. 3). Statistical analyses of the data show that across the Baltic catchments, N retention amounts to 72–88 % of NANI and P retention 85–96 % of NAPI. Knowing overall retention patterns for various watersheds is of obvious practical relevance, because this allows the country-wise required nutrient load reductions at the river mouth as formulated in the BSAP (HELCOM 2007) to be scaled to produce the required nutrient reductions at source, i.e., upstream in the watersheds. Overall, under the existing spatial distribution of nutrient loads across catchments, to reach the nutrient reduction goals of 135 000 tons N and 15 000 tons P as formulated in the BSAP from 2007 would require a reduction in nutrient loadings to the watersheds by 675 000 tons N and 158 000 tons P, respectively, assuming current estimates.

It is important to understand where nutrient retention is occurring within catchments in order to manage nutrient loads more effectively. MESAW, a statistical model developed by Grimvall and Stålnacke (1996) for source apportionment and retention of riverine loads of pollutants, has been applied to calculate nutrient retention (N and P) in surface water bodies within the Baltic Sea river basins. Input data consisted of land use (distinguishing cultivated areas, wetlands, lakes, and others), total drainage area, and point source emissions (from both waste water and industry). Results obtained for the same drainage basins as those used in the NANI/NAPI calculations have shown that the MESAW model was able to predict riverine loads of nitrogen very accurately; coefficients of determination between the observed and modeled data varied between 0.94 and 0.99. The estimated retention parameters were not statistically significant for the phosphorus model.

The MESAW calculations indicate that around 380 000 tons of nitrogen is retained annually in surface waters (streams, rivers, reservoirs, and lakes). In comparison, the total riverine load to the Baltic Sea for the 117 river basins was estimated to 570 000 tons N year^−1^, which amounts to an overall *surface* water nitrogen retention value of around 40 %. The three largest river basins (Neva, Vistula, and Oder) accounted for 50 % of the total retention. Results for phosphorus indicate retention of 12 000 tons compared to an estimated river load of 18 000 tons P year^−1^ for 76 Baltic drainage basins with measured P load, and thus an overall surface water P retention that is also around 40 %, but these P results are highly uncertain and should be used with caution. The values of nutrient retention in surface waters are understandably lower than the total catchment retention estimated from the NANI and NAPI analyses. Together, the NANI budget approach and the MESAW approach indicate that half of the total N retention on a Baltic-wide catchment scale occurs in surface waters, i.e., in watercourses and lakes, whereas the residual losses occur in groundwater and soils. For P, an even higher amount is retained in soils and groundwater, but estimates of surface water retention remain uncertain.

### Budget Calculations and Scenarios for Future Loads

**Fig. 4:**
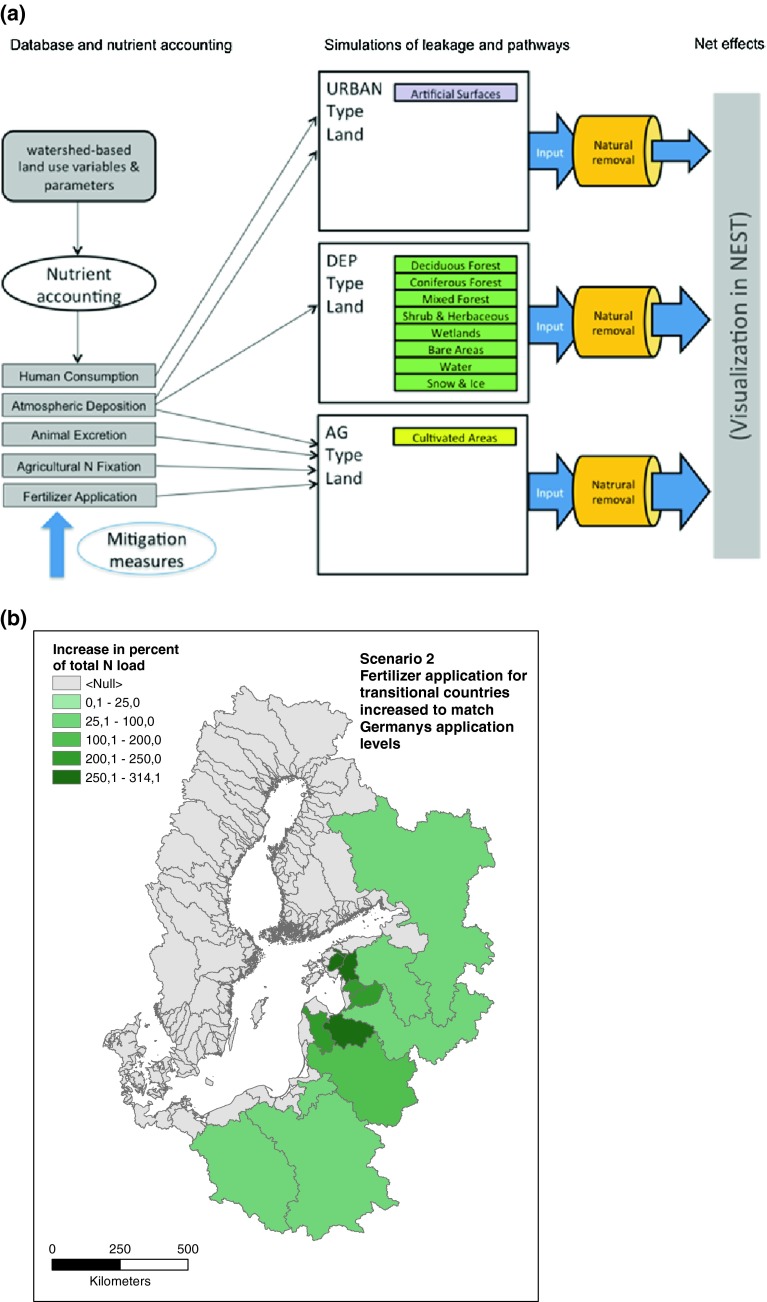
**a** Conceptual modeling framework linking the NANI budgets to a hydrological CSIM model allowing scenario analyses showing in **b** the potential effect of increased fertilizer use in transitional countries to levels as applied in Germany

The NANI budget calculations have been coupled to the catchment model CSIM as run in the NEST decision support system in order to undertake scenario analyses of possible future nutrient loads to the Baltic Sea. The various NANI components were distributed to CSIM land use categories (Fig. 4a). Such models have been used for exploring the effect of, e.g., different agricultural practices (BNI 2007; Hägg et al. 2010). With the new databases and models described here, we explored a possible scenario where fertilizer use in the transitional countries (Poland, Russia, and the Baltic States) increased to the levels now used in Germany, using overall nutrient retention estimates from the NANI approach and hydrological characteristics of the individual watersheds as described in CSIM. Modeling suggests that nutrient loads to the Baltic may increase drastically under this scenario (Fig. 4b). This scenario suggests that changes in agricultural practices in transitional countries could indeed have dramatic effects on the Baltic Sea ecosystem and the results also indicate which individual watersheds are most sensitive to these changes.

### Detailed Simulations of Nutrient Pathways and Effects of Abatement Measures for the Baltic Sea

Beyond estimating watershed nutrient retention, a dynamic model with much higher resolution approaching farm scale has been used to quantify the effects of various abatement measures on nutrient loads to the sea. NANI/NAPI calculations and the MESAW model cannot describe the detailed effects of changing management activities, e.g., in agriculture because they operate at broad spatial resolutions.

Abatement measures simulated were changes in fertilizer use, livestock density and atmospheric deposition, creation of wetlands, and improved sewage treatment. To quantify the effects of these measures, it is necessary to describe load reductions at various sources, as well as nutrient removal along transport pathways in soils, groundwater, lakes, and streams. Such estimates are also essential to calculate the cost-effectiveness of abatement measures, as described below.

To characterize important variables at a close to farm-level resolution, the Baltic Sea catchment (1.7 × 10^6^ km^2^) was partitioned into 10 × 10 km grid cells in which land use and agricultural practices were specified, based on information from a comprehensive dataset combining national and regional statistics and published surveys. The strong heterogeneity in farm size and production intensity within the catchment was characterized by introducing three different representative farm types, for which livestock, and fertilizer and manure inputs per crop were specified for each riparian country and calibrated to national statistics on consumption of fertilizer and manure. Within each country, the farm types were distributed at the NUTS2/Oblast/Voblast spatial scale using livestock production as a key, forming a consistent and very detailed description of agricultural production throughout the Baltic Sea catchment. Once the near farm-level variations in key variables were established, the soil–vegetation–atmosphere model DAISY was applied to estimate nutrient fluxes corresponding to the high-resolution farm management data (see Electronic Supplementary Material).

The high-resolution data in the Baltic Sea database enabled us to provide the DAISY model with information over the entire catchment, including precipitation, temperature, soil types, farm types, and levels of inputs of fertilizer and manure to crops. It thus became possible to describe relationships between these drivers and nutrient leaching for the entire region. These model outputs regarding catchment and soil type-specific fertilizer and manure inputs to crops were subsequently used as inputs to the cost modeling. Due to computational constraints, a direct link between DAISY and the cost-minimization model was not possible.

**Fig. 5:**
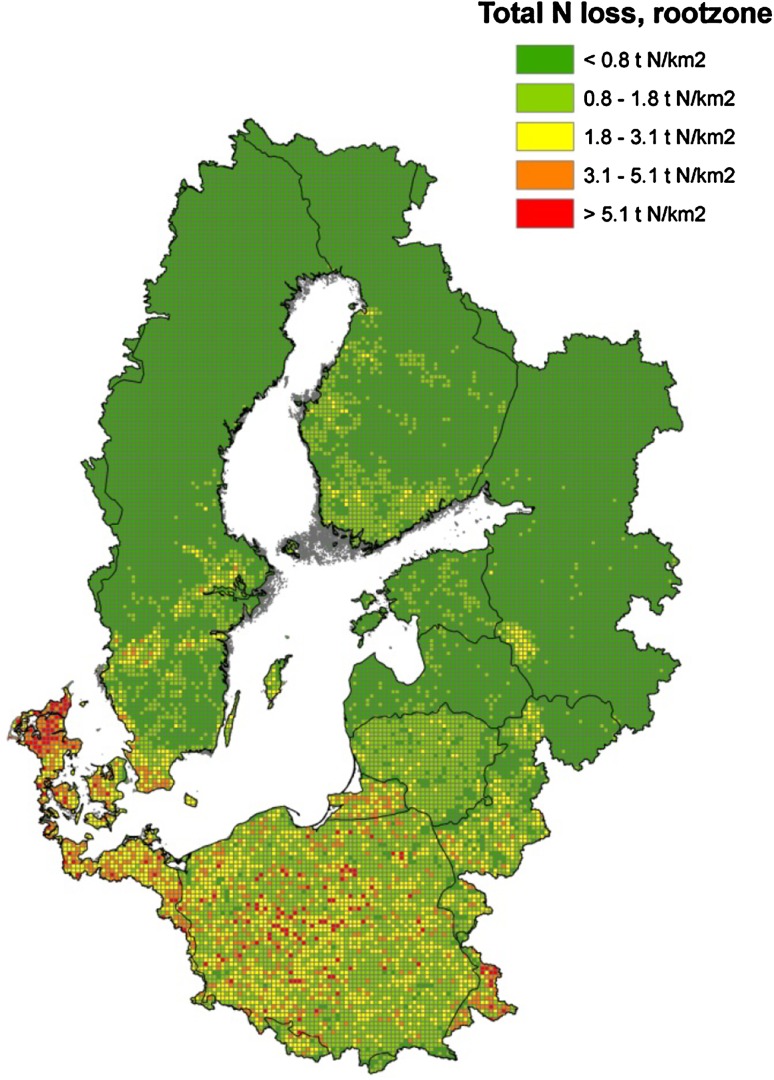
N leaching from the root zone (tons N km^2^) mapped on a 10-km grid level

Using simple multivariate N leaching functions derived from a summary of the Daisy model outcomes (see Electronic Supplementary Material) and the datasets on the characteristics of the Baltic catchments, we estimated N losses for the entire Baltic Sea catchment, including the effects of abatement measures. This enabled us to identify agricultural hot spots of nutrient emissions in a consistent way and at a level of detail not hitherto seen for this area (Fig. 5). The results of these calculations as well as the modeled retention described above (MESAW) were then used in the cost-effectiveness optimization.

**Fig. 6:**
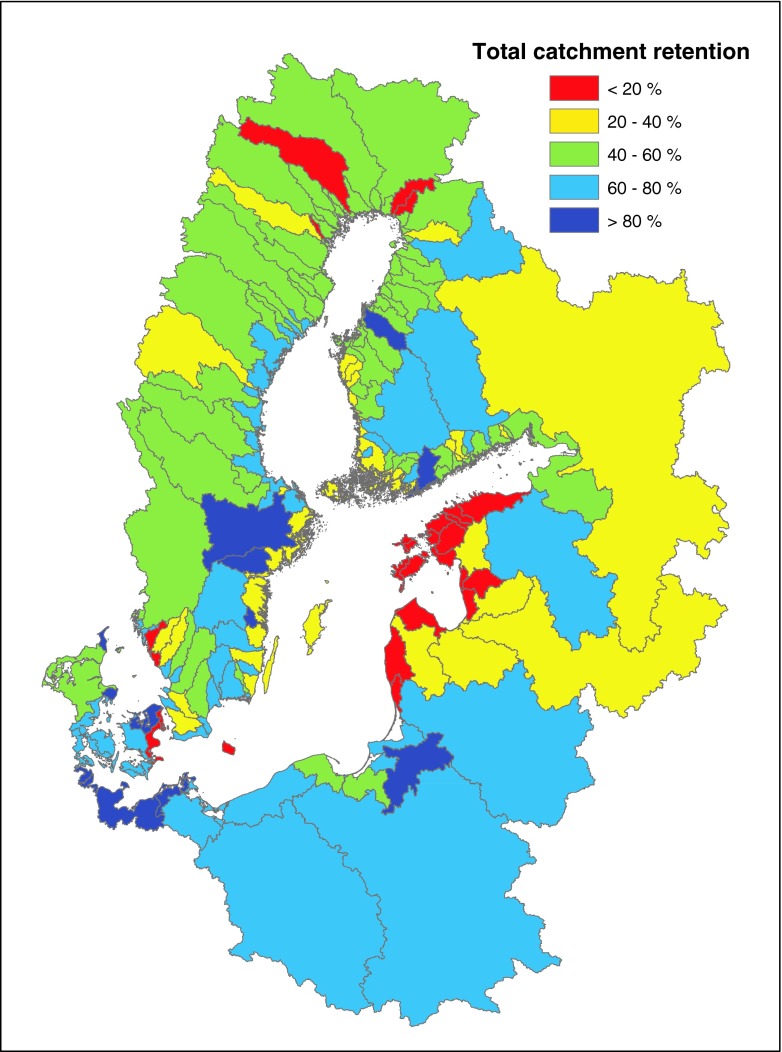
Total catchment N retention for 117 catchments draining to the Baltic Sea calculated by combining the results from the MESAW and DAISY models

**Fig. 7:**
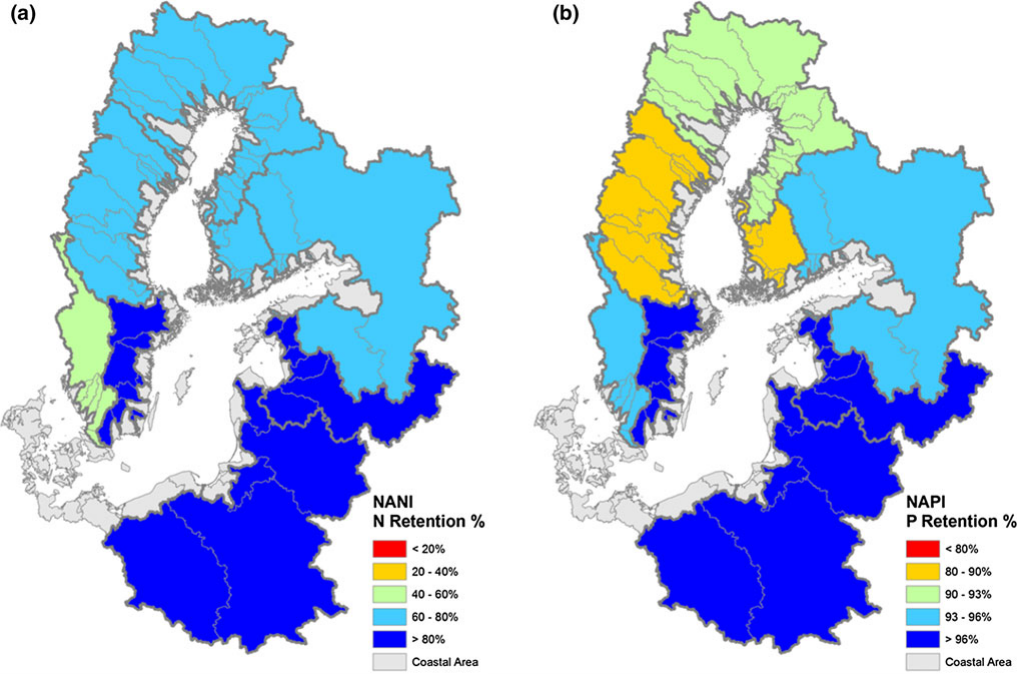
Regional N and P retention in the Baltic Sea basins (redrawn from Hong et al. 2012)

Overall nitrogen retention per catchment was estimated as the difference between riverine N losses and catchment root-zone N leaching, taking point sources into account (Fig. 6). Thus, retention in ground water and surface waters is included in this estimate, whereas retention in soils is already included in the estimate for root-zone N leaching. This approach was chosen because retention in ground water and surface waters at the catchment scale can be considered relatively constant, whereas retention in soils (including NH_3_ evaporation, denitrification, and N-sequestration in soil N pools) is highly variable within catchments and a function of, e.g., soil type, organic matter content, and applied N fertilizer (amount, type, application method, and timing of application). Consequently, soil retention was dealt with by the DAISY model and subsequently the N leaching function. Agricultural N losses are often estimated at the root-zone level since this allows comparisons between catchments and regions with different hydrological pathways and thus with different retentions (e.g., Andersen et al. 1999; Kronvang et al. 2008). Independent estimates of surface water retention by the MESAW model allowed us to split overall catchment N retention into estimates for groundwater and surface water retention. Regional N and P retention in the drainage basins estimated from the NANI and NAPI approach, respectively, showed a similar spatial pattern (Fig. 7).

### Abatement Cost Minimization

Together with costs and removal effectiveness at the source, removal of nutrients along the flow path through groundwater and surface waters (retention) determines the cost-effectiveness of nutrient abatement measures and the total costs of achieving specified coastal load reduction targets for the Baltic Sea sub-basins. The BALTCOST cost-minimization model (Hasler et al. 2012) has been developed to assess the selection and distribution of cost-effective nutrient reductions to the Baltic Sea which aim to fulfill the BSAP nutrient reduction targets (see also Electronic Supplementary Material). Various cost-minimization estimates are available in the scientific literature (Schou et al. 2006; Gren 2008; Elofsson 2010b), but this project provides more consistent estimates of the cost of undertaking different abatement measures across different regions. This represents a radical improvement of the estimates of the associated impact of these abatement measures on coastal nutrient loads as well as a detailed analysis of the capacity for implementing the different measures in different parts of the catchments. We now have access to far more detailed databases of human activities in the catchments as well as estimates of nutrient reductions related to these, i.e., through the DAISY model. All cost data are updated to the same year as the agricultural production data (2005). Thus, the BALTCOST cost-minimization model is based on far more accurate and consistent data than that available previously (see table in Electronic Supplementary Material).

The BALTCOST model uses separate coastal load reduction targets for N and P for the 7 Baltic Sea sub-basins (see Electronic Supplementary Material). BALTCOST seeks to identify the minimum-cost combination of N and P abatement measures across the catchments that drain into a particular sea sub-basin, subject to satisfying the reduction targets for *both* N and P loads into that particular sea sub-basin. Abatement cost minimization is carried out separately for each Baltic Sea sub-basin in turn to produce a cost-efficient solution for the Baltic as a whole, given the N and P load reduction targets that the BSAP assigned to the separate Baltic Sea sub-basins. BALTCOST does not account for nutrient transport between sea sub-basins or sources and sinks of nutrients internal to the sub-basins themselves, as these were already considered when the BSAP targets for nutrient reductions were set (HELCOM 2007).

The nutrient reduction measures included in BALTCOST are

- reductions in fertilizer applications to arable crops (N abatement)
- catch crops in spring-sown cereals (N abatement)
- reductions in livestock numbers (N & P abatement)
- restoring wetlands on agricultural land (N & P abatement)
- improving WWT (N & P abatement)

In the present BALTCOST model, livestock reductions are anticipated to have effects on both N and P abatement in most cases. It would be much harder to achieve the BSAP P load reduction targets, particularly in the Baltic Proper sub-basin if the effects of livestock reduction were excluded. More measures will be implemented in the BALTCOST model in the future, for instance, constructing wetlands on non-agricultural land, NO_*x*_ reductions from power plants and ships, as well as measures for increased utilization of livestock manure to decrease nutrient loads from livestock farms in those countries where the utilization rate is currently low (50 % of the nutrient content utilized or lower).

The BALTCOST model identifies the least-cost combination of the five nutrient abatement measures within each of 22 main Baltic drainage basins that will deliver the separate BSAP N and P load reduction targets specified for each of the seven Baltic Sea sub-basins.

The modeled capacity of the abatement measures, i.e., the maximum extent to which the measures can be implemented in each catchment, cannot be measured with full certainty. For example, the maximum allowable wetland restoration is estimated by mapping the share of organic agricultural soils within each catchment, and, consequently, a rather large share of the catchment can be converted into wetlands if the organic share is high and vice versa (wetland restoration capacity varies between 0.1 and 15 % of the agricultural land within the 22 drainage basins). We considered this approach to be less uncertain than assuming a fixed capacity constraint for wetland restoration for all catchments. A capacity constraint for the maximum reduction in nitrogen fertilizer application was set at 20 %. This constraint setting was chosen because reduction beyond this range is likely to influence the parameters of the yield functions from which the opportunity costs of fertilizer reduction were calculated. Increasing the maximum fertilizer reduction capacity beyond this 20 % limit could thus lead to faulty results as the shape of the yield functions will change due to depletion of the nitrogen stock in the soil. The maximum capacity for the livestock reduction measures was set at 30 % of the current herd sizes for the species concerned in each drainage basin. This capacity limit was chosen because further reductions in livestock numbers would be likely to incur additional costs, which are not reflected in the model, such as costs arising from prior investment in animal housing facilities and milking parlors. The reduction in manure fertilizer application to the field following livestock reductions is assumed to be substituted by commercial fertilizer with a lower nutrient loss. The application capacity constraint for catch crops is assumed to be the current land area sown with spring cereals, and for WWTP drainage basin-specific capacity constraints for WWT upgrading are estimated for each watershed, using data detailing the current implementation of WWT technology within the drainage basin (Berbeka et al. 2012).

The results in Table 2 report the minimum-cost combination of N and P abatement measures identified by BALTCOST at drainage basin spatial resolution for delivering load reductions that match the BSAP load reduction targets as fully as possible, given that the measure-specific maximum abatement capacities are implemented. BALTCOST results indicate that the BSAP load reduction targets can be delivered in all instances except for N reductions in the Danish Straits and P reductions in the Baltic Proper.

**Table 2:** Maximum load reduction targets for N and P, which could feasibly be delivered with the abatement measures so far implemented in BALTCOST

Sea region ID	N load reduction target (tons)	P load reduction target (tons)
Bothnian Bay	0	0
Bothnian Sea	0	0
Baltic proper	94 000	9290 (74 % of BSAP)
Gulf of Finland	6000	2000
Gulf of Riga	0	750
Danish Straits	13 120 (88 % of BSAP)	0
Kattegat	20000	0
Total	133 120	12 040

The lowest-cost combination of drainage basin-specific abatement measures, which almost achieves the BSAP load reduction targets (Table 2), delivers N and P reductions that exceed the targets specified for N for the Baltic Proper and the Gulf of Finland because some measures (livestock reductions, wetlands, and WWT upgrading) are anticipated to deliver both N and P reductions simultaneously. Thus, for example, increasing implementation of the wetlands’ measure to satisfy a P load reduction target will also deliver N load reductions, whether or not these N load reductions are required and vice versa.

**Fig. 8:**
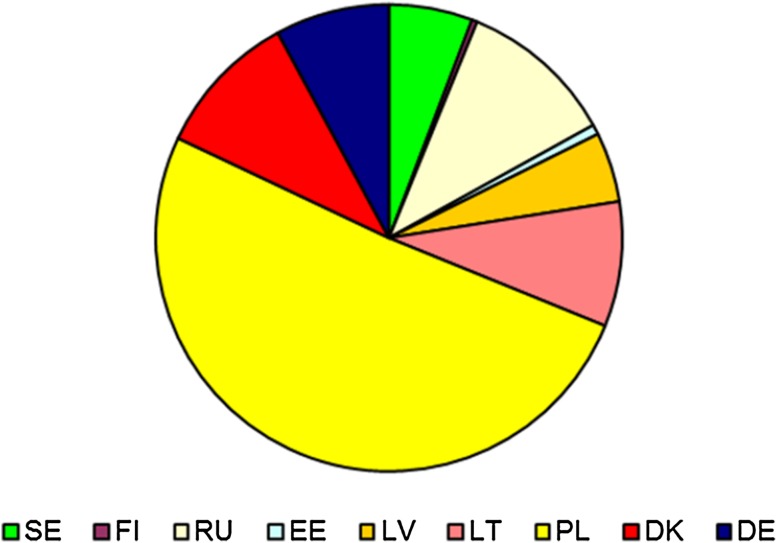
Distribution of the total annual costs of delivering the nutrient reduction targets among countries using the lowest-cost combination of drainage basin-specific abatement measures. Sweden (*SE*), Finland (*FI*), Russia (*RU*), Estonia (*EE*), Latvia (*LV*), Lithuania (*LT*), Poland (*PL*), Denmark (*DK*), and Germany (*DE*)

**Fig. 9:**
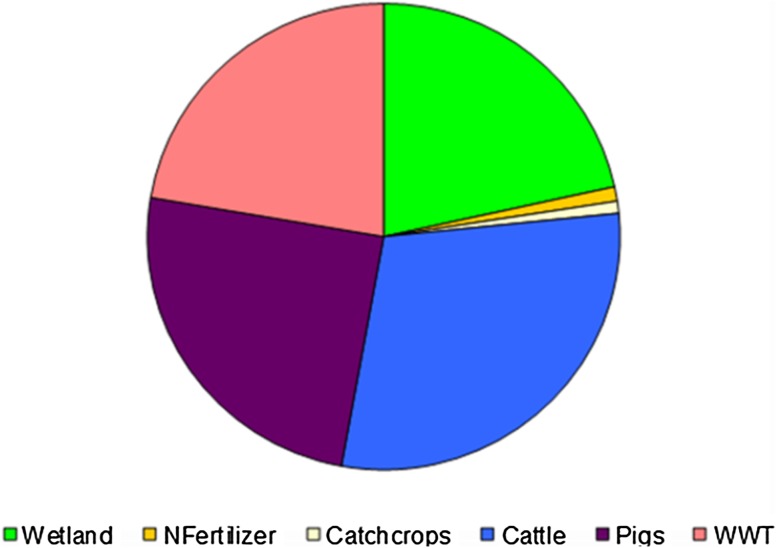
Distribution of the total annual costs of delivering the nutrient reduction targets between abatement measures using the lowest-cost combination of drainage basin-specific abatement measures

Figure 8 shows the total annual cost of delivering the load reduction targets using the lowest-cost combination of drainage basin-specific abatement measures distributed among countries. Figure 9 shows the distribution of total annual costs between abatement measures.

Total abatement costs are estimated to be 4.65 billion EUR annually, and a large part of these costs is incurred in Poland. This does not mean, however, that the costs should be borne by the citizens in Poland as the costs could potentially be distributed among the countries around the Baltic. Analyses of such cost-sharing schemes are outside the scope of this article, however.

**Fig. 10:**
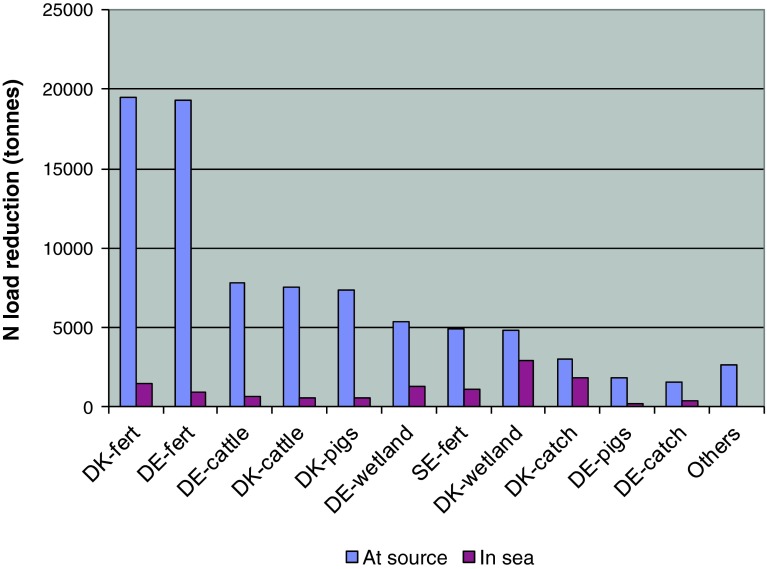
Comparison of N abatement at source with N load reductions achieved in the Danish Straits (at sea) by Denmark (*DK*), Germany (*DE*), and Sweden (*SE*) from the various N abatement measures

As mentioned earlier, there are problems in achieving the load reduction targets in the Baltic Proper and Danish Straits sub-basins. Delivery of N reduction in the Danish Straits to the level shown in Table 2 requires implementation of all measures with a capacity to reduce nitrogen. Figure 10 illustrates and compares N abatement at source and at sea between abatement measures in the lowest-cost combination of drainage basin-specific abatement measures, which delivers near-BSAP load reductions in the Danish Straits. Figure 10 clearly illustrates the considerable effect of N retentions in groundwater and surface water; abatement measures have to be implemented at very high intensity at source in order to deliver near-desired N load reduction in the receiving Baltic Sea sub-basin.

If retention was ignored, the costs of fulfilling the targets would be predicted (incorrectly) to be only 0.7 billion € per year, i.e., cost would be drastically underestimated.

### Socio-Economic Relevance and Policy Implications

Several problems have to be solved before a successful implementation of a cost-efficient policy that aims to meet the BSAP nutrient reduction targets can be achieved. First, it is a difficult task to identify a distribution of the cost-effective abatement burden on which all countries will agree. The difficulty lies in the fact that costs are not written in stone, but can change over time in response to changes in technologies and consumer demand. Moreover, and particularly relevant for the Baltic Sea, countries with many low-cost or high-capacity abatement options may also be those with lower technological and institutional ability to deliver abatements. These countries may also show a lower social willingness to pay, perhaps as a consequence of the national population deriving lower social benefits from improvements in the ecological condition of the Baltic Sea (Markowska and Zylicz 1999; Ollikainen and Honkatukia 2001; Ahtiainen 2009). It is well known that these problems can be relieved to some extent by international emissions trading, where distributional concerns can be solved through allocation of emission permits, an issue which has attracted much attention in the climate policy context (Rose et al. 1998). Elofsson (2010b) shows that if applied at the Baltic-wide level, nutrient emission trading could potentially solve part of a problem that arose when the BSAP nutrient load reduction targets were developed without explicit consideration of equity concerns. A full-scale decentralized emission-trading scheme for the whole Baltic Sea basin would require trading ratios to be defined for measures of different types and in different locations. Such trading ratios could, in principle, be derived from the BALTCOST model. Yet, such a scheme is likely to be associated with substantial transaction costs, i.e., costs for administration and enforcement. However, emission trading need not require that all sectors and sources trade in a decentralized manner (compare, for instance, with the EU carbon dioxide emission-trading scheme, where only a subset of the emitting sectors is included).

Although emission trading could to some extent provide a mechanism for implementing a cost-efficient distribution of nutrient abatement efforts at an international level, much of the implementation is currently undertaken at the regional and local level—and it is likely to remain so. The distribution of efforts across governments at different levels occurs for several reasons, including the large variety of emissions sources, the combination of large-scale environmental degradation with locally severe environmental problems, and institutional practice with regard to the implementation of different kinds of policy instruments in different sectors. As shown in Elofsson (2011), such a multi-level governance system can give rise to unwanted strategic behavior by the governments involved, with higher-level governments potentially attempting to shift costs away from their own budgets and onto the budgets of lower-level governments. One way to reduce the incentives for such strategic behavior is to change the allocation of decision rights over different nutrient abatement policies, either through increased centralization or decentralization. Elofsson (2011) shows that local governments can have economic justifications for avoiding decentralization of policies for wetlands even if they have better knowledge of the suitable design of wetlands and if decentralization is advantageous to society overall. Local governments would be less reluctant to take on additional regulatory responsibilities if they could stipulate conditions for decentralization, and if they were already in charge of other nutrient policies such as those regulating local wastewater, factors that could also improve the socio-economic outcome. Reconsideration of the currently disjointed policymaking with regard to the potential benefits of centralization or decentralization should acknowledge strategic incentives as well as accounting for the potential effect that intergovernmental grants could have on these incentives.

## Discussion and Conclusions

Today, diffuse losses from agriculture are the most important nutrient sources to the Baltic Sea; this has become even more evident during recent decades as point source effluents continue to decrease due to improved sewage treatment. It is unlikely that the preconditions for good environmental status of the Baltic Sea will be achieved by 2021 if the nutrient reduction targets from only municipal wastewaters are fulfilled (Wulff et al. 2007; HELCOM 2012).

Climate change is expected to lead to intensified agriculture in the northern regions of Europe to compensate for projected production losses in southern Europe (Olesen and Bindi 2002; Maracchi et al. 2005). Agriculture in Eastern Europe is still less intensive than in Western European countries. Our model simulations indicate a massive increase in fertilizer use within the Baltic catchment if the agricultural practices of Western Europe are applied everywhere (Fig. 7). The anticipated intensification of agricultural production may lead to increased nutrient leakages, nullifying the gains of improved sewage treatment. The scenario presented here illustrates not only the importance of developing measures to reduce loads from agriculture but also the strong heterogeneity in agricultural practices, soil types, and hydrology within the region. The most cost-effective measures in, for instance, Denmark, may not be relevant in Poland. The databases and models described here should be useful in further discussions among stakeholders, managers, and scientists in search of a sustainable productive agricultural system while still reducing nutrient loads and restoring the Baltic Sea.

The BALTCOST model supplements existing cost-modeling tools for the Baltic, as it is built on intensive, detailed interdisciplinary work and data exchange between natural scientists and economists within the RECOCA project. This economic model indicates that it is possible to achieve the BSAP load reduction targets for N and P in most Baltic Sea regions, with the exception of the P load target in the Baltic Proper and the N reduction target in the Danish Straits, where only 74 and 88 % respectively, of the desired BSAP load reductions can be delivered within modeled abatement capacity constraints. The minimized total cost per year of delivering these near-BSAP load reductions across the 9 Baltic littoral countries is estimated to be 4.69 billion €. This result is comparable with the cost predictions produced in previous studies. For example, Elofsson (2010b) concluded that previous models predicted costs of 2.6–5.0 billion € per year to deliver the load reductions specified in the BSAP. The differences between the cost predictions of the models can be explained by variations in the number of abatement measures included, differences in the modeled cost estimates, differences in the implementation of the load reduction targets, and—particularly important in a RECOCA context—differences in the spatial resolution of the modeled nutrient retention. Our results show that differences in the way in which retention is modeled can exert considerable influence over the minimum predicted abatement costs. Retention varies not only between catchments but also within catchments depending on hydrological pathways (e.g., between tile drained and non-tile drained areas). Thus, future research into identification and mapping of areas with low retention could further stimulate application of cost-effective measures.

These models can be used to explore comparisons of nutrient management strategies between different regions, which could be relevant for policy analysis of coastal load reductions to the entire Baltic. It is obvious that the country allocation scheme that is used now, based on the principle of “polluters pay,” is far more expensive than a scheme based on cost minimization, but implementing such a minimum-cost scheme would require some form of compensation or a nutrient-trading system to be adopted on at least a regional scale. Nutrient trading has been implemented in other regions, particularly in the US (EPA 2013). For the Baltic Sea, the trading system would necessarily be international, and realization of such a system is unlikely in the immediate term (Ollikainen and Honkatukia 2001). Regulatory disincentives have frozen many water quality-trading initiatives, especially those involving nonpoint sources (King 2005). Nutrient trading requires decisions on the extent to which, and where, excessive nutrient loads should be eliminated; in a Baltic context, this is defined by the BSAP. As pointed out by, e.g., NEFCO (2008), a prerequisite for successful implementation of a nutrient-trading regime is the availability of good data on the costs and effectiveness of abatement measures undertaken at different locations. The studies reported here clearly show that we are rapidly closing the gap between nutrient-trading policy and science.

## Electronic supplementary material

Below is the link to the electronic supplementary material.

Supplementary material 1 (PDF 124 kb)
