# Supplementary material for: Reduction of Baltic Sea Nutrient Inputs and Allocation of Abatement Costs Within the Baltic Sea Catchment
Source: Ambio. 2014 Jan 12;43(1):11–25. doi: 10.1007/s13280-013-0484-5 (PMC3888655; doi:10.1007/s13280-013-0484-5)
Supplement: Supplementary file 1 — Supplementary material 1 (PDF 124 kb) [file 13280_2013_484_MOESM1_ESM.pdf]

Electronic Supplementary Material:

Reduction of Baltic Sea Nutrient Inputs and Allocation of Abatement Costs within the Baltic Sea Catchment

Fredrik Wulff, Hans Estrup Andersen, Gitte Blicher-Mathiesen, Mikołaj Czajkowski,Katarina Elofsson, Anders Fonnesbech-Wulff, Berit Hasler, Bongghi Hong, Christoph Humborg, Viesturs Jansons, Carl-Magnus Mörth, James C.R. Smart, Erik Smedberg,Per Stålnacke, Dennis P. Swaney, Hans Thodsen, Adam Was and Tomasz Żylicz.

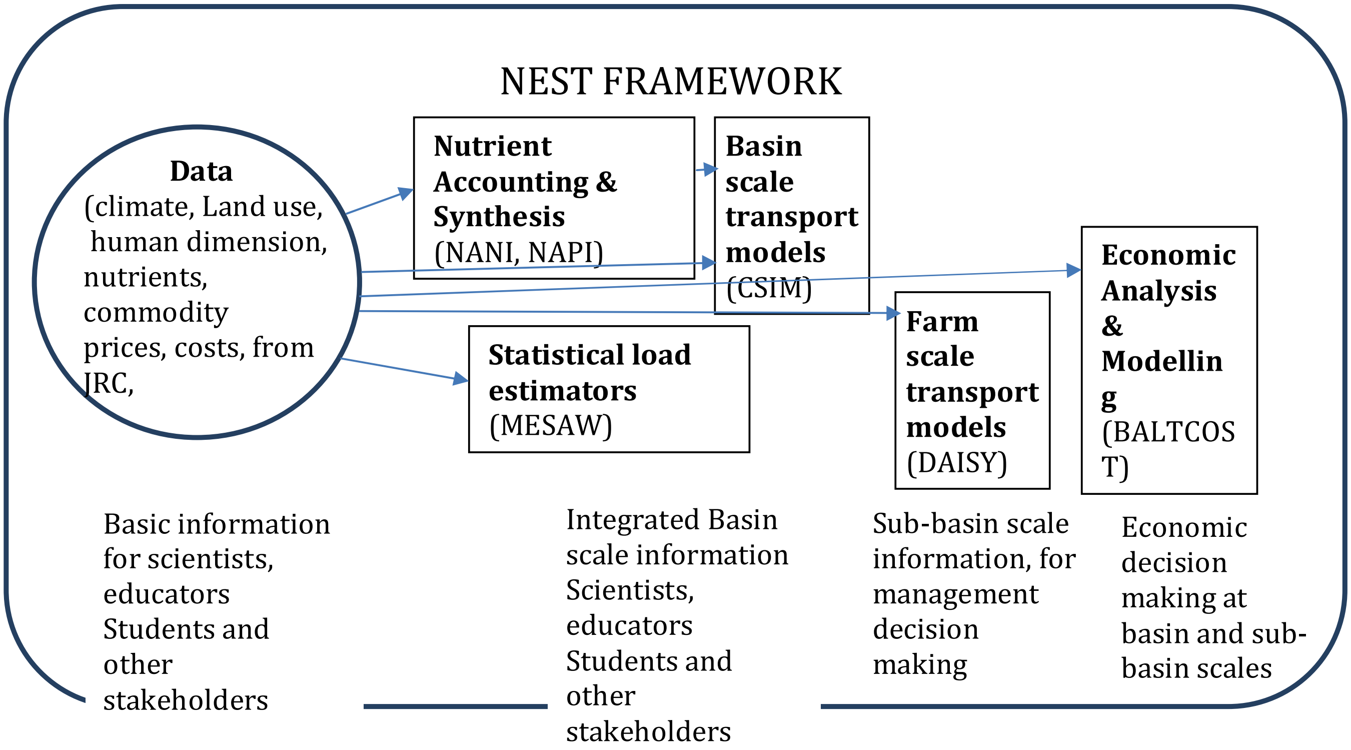

Fig. S1 Hierarchy of tools used for Baltic water quality management within NEST. Examples of tools used at each level are indicated in parentheses. Several levels of analysis and information delivery are involved in watershed management, each with their own requirements and levels of complexity appropriate to the task at hand. Level of complexity and spatio-temporal resolution tends to increase from left to right, as do the requirements of data and parameter estimation, and thus the level of uncertainty. Balancing uncertainty against demands for information for each purpose is central to the multi-model approach, and comparison of different model approaches should lead to more robust management.

Model summaries

NANI and NAPI toolbox

Net anthropogenic nitrogen input (NANI) is calculated as the sum of four major components: atmospheric N deposition, fertilizer N application, agricultural N fixation, and net food and feed imports, which in turn are based on the balance of crop and animal N production and animal and human N consumption. Nitrogen in food, consumed in excess of supply within a watershed is assumed to be supplied by imports; supply in excess of demand represents an export from the watershed. We created a NANI toolbox that extracts and compiles the GIS data relevant for nutrient budgets from the RECOCA database at the watershed level (Hong et al. 2011). A similar accounting approach has also been applied to phosphorus, resulting in the calculation of NAPI (Net Anthropogenic Phosphorus Input) (Han et al. 2011; Russell et al. 2008). Hong et al. (2012) developed a new version of the NANI and NAPI toolbox, which incorporates all of these improvements for the Baltic Sea catchments.

MESAW

MESAW is a statistical model for source apportionment of riverine loads of pollutants developed by Grimvall & Stålnacke (1996). This model-approach uses non-linear regression for simultaneous estimation of e.g. source strength, i.e. export coefficients to surface waters, for the different specified land cover or soil categories and retention coefficients for pollutants in a watershed. MESAW provides the possibility to calculate source apportionment and retention of nutrients in a large drainage basin characterized as both data-rich and data-poor. Based on knowledge about water quality, as well as geographical information on factors such as land-use, soil type, vegetation, lake area etc. the inputs and outputs are linked and the most likely sources are estimated. More specifically the model assumes that pollutant loads from a catchment area to a lake is a function of a "production term" related to land cover; emissions, and "reduction term" related to processes in the catchment that store or remove pollutants; retention. The MESAW model uses nutrient loads for a fixed time interval at each monitoring

site as the response variable and the characteristics of basins as explanatory variables to estimate diffuse nutrient emissions through non-linear regression analysis. Examples of practical application of the MESAW model are given in Liden et al. (1999), Vassiljev & Stålnacke (2005), Vassiljev et al. (2008) and Povilaitis et al (2012).

The CSIM watershed model

CSIM is a lumped, watershed-scale hydrological model based on the generalized watershed loading function model (GWLF, Haith and Shoemaker 1987). Variants of these models have been used successfully in several watersheds in the United States (Dai et al. 2000; Lee et al. 2000, 2001; Schneiderman et al. 2002; Hong and Swaney 2004), and elsewhere (Ning et al. 2002; Hristov et al. 2004; Smedberg et al. 2006; Sha et al. 2013) including the Baltic catchment (Mörth et al. 2007) to simulate seasonal and inter annual nutrient fluxes. The model divides each watershed into land use categories and considers the loads from each category separately. It is based on characteristic concentrations of inorganic and total N and P in surface and ground waters.

DAISY and the development of a summary N loss function

DAISY is a soil–plant–atmosphere system model designed to simulate water balance, heat balance, solute balance and crop production in agro-ecosystems subjected to various management strategies (Hansen et al. 1991; Abrahamsen and Hansen 2000). The soil-water balance includes water flow in the soil matrix as well as in macro-pores. It also includes water uptake by plants and a model for drainage to pipe drains. The solute-balance model simulates transport, sorption and N transformation processes including mineralization, immobilization, nitrification and denitrification, sorption of ammonium, uptake of nitrate and ammonium, and leaching of nitrate and ammonium. DAISY has been applied in several settings in Europe (e.g. Hansen et al. 2001; Heidmann et al. 2008). The model was calibrated to monitored root-zone N losses in mini catchments and to regional and national statistical data on crop yields.

Quantifying the effect of eco-engineering approaches such as wetland formation was based on existing experimental data achieved by consulting national experts. Wetland retention was modeled as rate constants: restored wetlands 150 kg N ha-1 yr-1 and 0.7 kg P ha-1 yr-1, and constructed wetlands 300 kg N ha-1 yr-1 and 1.4 kg P ha-1 yr-1 .

The high resolution data in the Baltic Sea database provide the DAISY model with sufficient information (including precipitation, temperature, soil types, farm types and levels of inputs of fertilizer and manure to crops) to simulate responses to the combinations of environmental conditions occurring over the entire region (more than 11,000 combinations), thus making it possible to describe the relationship between these drivers and nutrient leaching for the entire region over the period of available climate data (1995 – 2006). Based on the outcomes of these DAISY simulations, we identified the most significant variables by the use of covariance analysis with the parameters estimated using a least squares method (Rawlings 1988) and developed a multivariate statistical N leaching function for N losses from the root-zone of agricultural land in the Baltic Sea catchment. The function describes how agricultural management interacts with natural physiography and calculates agricultural N losses as a function of crop type (variable Crop, 14 different types possible), total N input to the crop including fertilizer, manure, N-fixation, N in seeds, and atmospheric N deposition (variable Total N, units kg N ha-1 yr-1), agricultural management (variable Farm, comprising three farm types (arable, intensive livestock, extensive livestock)), clay content in the topsoil (0-30 cm) (variable Clay, units weight percent) and carbon content in the topsoil (0-30 cm) (variable Clay, units weight percent):

$$\mathrm{N\,loss} = \beta_0 \cdot \exp\left(\mathrm{Crop} + \mathrm{Farm} + \beta_1 \cdot \mathrm{Clay} + \beta_2 \cdot \ln\left(\mathrm{Total\,N}\right) + \beta_3 \cdot \mathrm{Carbon}\right)$$

R2 = 0 .91

Where N-loss is N leaching from the root-zone (kg N ha-1 yr-1) and β_0 - β_3 are model parameters.

THE BALTCOST economic model

The BALTCOST model (Hasler et al. 2012) uses separate coastal load reduction targets for N and P for the 7 Baltic Sea sub-basins. BALTCOST seeks to identify the minimum-cost combination of N and P abatement measures across the catchments that drain into a particular sea sub-basin, subject to satisfying the reduction targets for both N and P loads into that particular sea sub-basin. Input data are described in Table S1.

Cost functions, effect functions, capacity constraints (which dictate the maximum extent to which abatement measures can be implemented) and catchment-scale nutrient retentions are calibrated using relevant combinations of data at national, watershed and 10 x 10 km resolution scale, thereby using the disaggregated data from the other components of the RECOCA project. The optimization problem can be formulated as minimizing the total cost of achieving the BSAP load reduction targets:

$$min\,TC = \sum_{DB}\sum_{m=1}^{m=6} C_{DB,m}\left(a_{DB,m}\right)$$

subject to the following constraints for each ($\forall$) of the seven Baltic sea sub-basins:

$$\forall_{SR=1..7} \sum_{DB\in SR}\sum_{m=1}^{m=6}\left(1-R_{DB}^{N_s}\right)\left(1-D_m R_{DB}^{N_g}\right)e_{DB,m}^{N}\left(a_{DB,m}\right) \geq T_{SR}^{N}$$

$$\forall_{SR=1..7} \sum_{DB\in SR}\sum_{m=1}^{m=6}\left(1-R_{DB}^{P_s}\right)\left(1-D_m R_{DB}^{P_g}\right)e_{DB,m}^{P}\left(a_{DB,m}\right) \geq T_{SR}^{P}$$

$$\forall_{DB=1..22}\ \forall_{m=1..6}\ a_{DB,m} \leq a_{DB,m}^{max}$$

where:

SR indexes 7 sea sub-basins,

DB indexes the drainage basins,

m indexes the 6 abatement measures,

N indicates nitrogen,

P indicates phosphorus,

$C_{DB,m}(\cdot)$ denotes a drainage basin specific cost function associated with implementing measure m at the level $a_{DB,m}$,

$e_{DB,m}(\cdot)$ denotes drainage basin specific effect functions for N and P, respectively, associated with implementing measure m at the level $a_{DB,m}$,

T_{SR} is the nutrient load reduction target allocated to a particular sea region for nitrogen (N) and phosphorus (P), respectively,

R_{DB} is the drainage basin specific surface (s) and ground (g) water retention for nitrogen (N) and phosphorus (P), respectively,

D_m is a binary 'switch' variable associated with whether a measure m abates nutrient emissions on the land surface ($D_m = 1$) or directly into surface waters ($D_m = 0$).

The BALTCOST model optimizes the implementation of abatement measures in each of the drainage basins to reach the targets specified for all the sea sub-basins. It is important to note that our approach utilizes retention coefficients and capacity constraints as well as cost and effect functions which are drainage basin specific – i.e. retention coefficients and capacity constraints were calibrated to each drainage basin using relevant combinations of data at national, watershed and 10 x 10 km resolution. The 6 measures and their effects are anticipated to be independent so that the effect of one measure will not be influenced by the implementation of another measure. This assumption holds for implementation of wastewater treatment and livestock reductions, but the other measures (catch crops, wetland restoration and fertiliser reductions) will be mutually dependent. This is a shortcoming of the model as applied, and further research is needed to estimate the effect of implementing these measures together, for instance the effects on nutrient reductions from wetlands when the nutrient transport through the wetland reduces, and the effect of catch crops when fertiliser application is reduced at the same time. These effects are not presently known.

The BALTCOST model can be used to estimate total abatement costs as well as marginal costs of abatement, as information about both total abatement costs and marginal costs is essential for policy advice.

References of supplementary material

Abrahamsen, P. and S. Hansen. 2000. Daisy: An Open Soil-Crop-Atmosphere System Model. Environmental Modelling and Software 15(3): 313–330

Dai, T., R.L. Wetzel, R.L. Tyler and E.A. Lewis. 2000. BasinSim 1.0: A Windows Based Watershed Modeling Package. Virginia Inst. of Marine Science, College of William and Mary. Retrieved March 11, 2013, from http://www.vims.edu/bio/models/basinsim.html

Grimvall, A. & Stålnacke, P. (1996). Statistical methods for source apportionment of riverine loads of pollutants. Environmetrics, 7 (2): 201-213.

Haith, D.A. and L.L. Shoemaker. 1987. Generalized watershed loading functions for streamflow nutrients. Water Resources Bulletin 23: 471–478.

Han, H., N. Bosch, and J.D. Allan. 2011. Spatial and temporal variation in phosphorus budgets for 24 watersheds in the Lake Erie and Lake Michigan basins. *Biogeochemistry* 102: 45–58.

Hansen, S., H.E. Jensen, N.E. Nielsen, and H. Svendsen. 1991. Simulation of nitrogen dynamics and biomass production in winter wheat using the Danish simulation model Daisy. Fertilizer Research 27: 245-259.

Hansen, S., C. Thirup, J.C. Refsgaard, and L.S. Jensen. 2001. Modelling of nitrate leaching at different scales – application of the Daisy. In: Modelling Carbon and Nitrogen Dynamics for soil Management, ed. M. Shaffer, M. Liwang, and S. Hansen, pp. 511-547. Boca Raton: Lewis Publishers

Hasler B., J.C.R. Smart, and A. Fonnesbech-Wulff A. 2012. Structure of BALTCOST Drainage Basin scale abatement cost minimisation model for nutrient reductions in Baltic Sea regions Deliverable 8.1. RECOCA. Retrieved December 8, 2013 from http://nest.su.se/recoca/deliverable_8.1.pdf.

Heidmann, T., C. Tofteng, P. Abrahamsen, F. Plauborg, S. Hansen, A. Battilani, J. Coutinho, F. Doležal, et al. 2008. Calibration procedure for a potato crop growth model using information from across Europe. Ecological Modelling 211(1-2): 209-223.

Hong, B. and D.P. Swaney. 2004. GWLFXL Users' Manual. Cornell University. Retrieved February 11, 2013, from http://www.eeb.cornell.edu/biogeo/usgswri/GWLFXL/gwlfxl.doc

Hong, B., D.P. Swaney, C.-M. Mörth, E. Smedberg, H.E. Hägg and C. Humborg. 2011. NANI/NAPI Calculator Toolbox version 2.0 documentation: Net Anthropogenic Nutrient Inputs in Baltic Sea catchments. BNI technical report series, technical report 3, ISBN: 978-91-86655-02-0. Retrieved February 11, 2013, from http://www.balticnest.org/balticnest/research/publications/publications/naninapicalculatortoolboxversion20documentationnetanthropogenicnutrientinputsinbalticseacatchments.5.2beb0a011325eb5811a8000123394.html

Hong, B., D.P. Swaney, C.-M. Mörth, E. Smedberg, H.E. Hägg, C. Humborg, R.W. Howarth, and F. Bouraoui. 2012. Evaluating regional variation of net anthropogenic nitrogen and phosphorus inputs (NANI/NAPI), major drivers, nutrient retention pattern and management implications in the multinational areas of Baltic Sea basin. *Ecological Modelling* 227:117-135.

Hristov, T., B. Evans, and V. Ionecheva, V. 2004. Simulation of stream flow in the Yantra River Basin, Bulgaria via a GIS-based modeling approach. In: Transactions of the 2nd Biennial Meeting of the International Environmental Modeling and Software Society. Osnabruck, Germany, pp. 1123–1128.

Lee, K.Y., T.R. Fisher, T.E. Jordan, D.L. Correll, and D.E. Weller. 2000. Modeling the hydrochemistry of the Choptank River Basin using GWLF and Arc/Info: 1. Model calibration and validation. Biogeochemistry 49: 143–173.

Lee, K.Y., T.R. Fisher, and E. Rochelle-Newall. 2001. Modeling the hydrochemistry of the Choptank river basin using GWLF and Arc/Info: 2. Model validation and application. Biogeochemistry 56: 311–348.

Liden, R., Vasilyev, A., Stålnacke, P., Loigu, E. & Wittgren, H. B. (1999). Nitrogen source apportionment - a comparison between a dynamic and a statistical model. Ecological Modelling, 114 (2-3): 235-250.

Ning, S.K., K.Y. Jeng, and N.B. Chang. 2002. Evaluation of non-point sources pollution impacts by integrated 3S information technologies and GWLF modeling. Water Science and Technology 46: 217–224.

Mörth, C. M., C. Humborg, H. Eriksson, A. Danielsson, M.R. Medina, S. Löfgren, D.P. Swaney, and L. Rahm. 2007. Modeling riverine nutrient transport to the Baltic Sea: A large-scale approach. Ambio 36(2-3): 124-133.

Povilaitis, A., Stålnacke, P. and Vassiljev, A. (2012). Nutrient retention and export to surface waters in Lithuanian and Estonian river basins. Hydrology Research, 43(4):359-373.

Rawlings, J.O.1988. Applied Regression Analysis. Belmont, CA: Wadsworth and Brooks/Cole.

Russell, M.J., D.E. Weller, T.E. Jordan, K.J. Sigwart, and K.J. Sullivan. 2008. Net anthropogenic phosphorus inputs: spatial and temporal variability in the Chesapeake Bay region. Biogeochemistry 88: 285–304.

Schneiderman, E.M., D.C. Pierson, D.G. Lounsbury, and M.S. Zion. 2002. Modeling the hydrochemistry of the Cannonsville watershed with Generalized Watershed Loading Functions (GWLF). Journal of the American Water Resources Association. 38: 1323–1347.

Sha, J., M. Liu, D. Wang, D. P. Swaney, and Y. Wang. 2013. Application of the ReNuMa model in the Sha He river watershed: Tools for watershed environmental management. Journal of Environmental Management. 124:40-50, ISSN 0301-4797, 10.1016/j.jenvman.2013.03.030.

Smedberg, E., C.-M. Mörth, D.P. Swaney, and C. Humborg. 2006. Modeling hydrology and silicon-carbon interactions in taiga and tundra biomes from a landscape perspective: implications for global warming feedbacks. Global Biogeochemical Cycles 20(2): GB2014.

Vassiljev, A., Blinova, I. & Ennet, P. (2008). Source apportionment of nutrients in Estonian rivers. Desalination, 226 (1-3): 222-230.

Vassiljev, A. & Stålnacke, P. (2005). Source apportionment of nutrients in the Lake Peipsi drainage basin - experiences from a statistical model. Water Science, and Technology 51 (3-4): 309-317.

Table S1 Data inputs to the BALTCOST model.

**Data**	**Data source in and outside RECOCA**	**Resolution and unit**	**Purpose in BALTCOST**
Fertilizer use	EU JRC, EUROSTAT, FADN, DAISY model outputs	117 watersheds, at 10 x 10km resolution	N and P inputs per ha in agriculture in baseline (2005)
Fertilizer and manure use	DAISY model outputs	117 watersheds, at 10 x 10km resolution	Information about soil type-specific fertilizer and manure inputs to crops
Crop area	EU JRC, CORINE, EUROSTAT, FADN	117 watersheds, at 10 x 10km resolution	Crop production in baseline (2005), used for estimating opportunity costs of wetland restoration, catch crops and the costs of fertilizer reductions.<br><br>Used to estimate capacity limits for catch crops, as well as for fertilizer

			reductions.
Livestock	EUROSTAT, FADN	117 watersheds, livestock numbers, cattle/pigs at 10 x 10km resolution	Pig and cattle production in baseline (2005), used for estimations of costs of livestock reductions.<br><br>Used to estimations of capacity limits for reductions of livestock production.
Human population	HYDE	117 watersheds, population number at 10 x 10km resolution	Distribution and density of population, linked to information about existing WWTPs to estimate potentials for improved treatment and connection to treatment facilities.<br><br>Used to estimate capacity limits for improvements of wastewater treatment.
Wastewater treatment plants (WWTPs)	EUROSTAT, HELCOM & OECD	117 watersheds	Location, size and current level of treatment for existing WWTPs, used in conjunction with population distribution to determine maximum upgradable

			population size in each watershed.

Table S1 continued

**Data**	**Data source in and outside RECOCA**	**Resolution and unit**	**Purpose in BALTCOST**
Effects on leaching	DAISY	117 watersheds, per hectare at 10 x 10km resolution	Multivariate N leaching functions derived from a summary of the Daisy model outcomes, used to estimate N losses for each of the abatement measures. Soil retention.
Retention	MESAW	117 watersheds	Data used to for modeling groundwater and surface water retention of Nitrogen.
Soil types	EUROSTAT, CORINE	117 watersheds	Used for estimating capacity for wetland restoration. Used to estimate effect on load reduction from fertilizer reduction and catch crops.
Standard outputs, gross margins and prices for inputs and outputs in agriculture	EUROSTAT	NUTS 2 resolution	Used in conjunction with appropriate cost and profit functions to determine opportunity costs of reduced fertilizer

			applications or reduced livestock numbers
Dietary intake and excretion of nitrogen and phosphorus by human population	NANI/NAPI calculator toolbox	National	Used to calculate reductions in N and P loads at source following up-grading of WWTPs
Nitrogen and phosphorus excretion rates by cattle and pigs of different age classes	NANI/NAPI calculator toolbox	National	Used to calculate reductions in N and P loads at source following reductions in livestock numbers
Average crop yield by crop type	EUROSTAT & Belarus Department of Agriculture	National	Used to calculate opportunity cost of reduced fertilizer applications
Electricity prices	EUROSTAT & Russian electricity price report	National	Used to calculate wastewater treatment costs at national resolution
Labor wage rates	OECD & Russian wage rate report	National	Used to calculate wastewater treatment costs at national resolution
